# Development and validation of an integrated preclinical model mimicking cardiometabolic risk in postmenopausal female rats

**DOI:** 10.1038/s41598-025-26559-z

**Published:** 2025-11-27

**Authors:** P. Sainath, Shakta Mani Satyam, Sanjay Bharati, Prakashchandra Shetty, Abdul Rehman, Mohamed El-Tanani, Laxminarayana Kurady Bairy, Akheruz Zaman Ahmed, Tanya Densil, Safaa Fathima, Raya Famin Harid, Rashmi Kumari, Yahia El-Tanani, S. Ganesh Kamath, Guruprasad D. Rai

**Affiliations:** 1 Department of Perfusion Technology, Manipal College of Health Professions, Manipal Academy of Higher Education, Manipal, 576104 India; 2 Department of Pharmacology, RAK College of Medical Sciences, RAK Medical and Health Science University, Ras al Khaimah, 11172 United Arab Emirates; 3 Department of Nuclear Medicine, Manipal College of Health Professions, Manipal Academy of Higher Education, Manipal, 576104 India; 4 Department of Anatomy, Manipal University College Malaysia, Melaka, 75000 Malaysia; 5 Department of Pathological Sciences, College of Medicine, Ajman University, Ajman, 346 United Arab Emirates; 6 RAK College of Pharmacy, RAK Medical and Health Science University, Ras al Khaimah, 11172 United Arab Emirates; 7 Department of Anatomy, Fakhruddin Ali Ahmed Medical College, Srimanta Sankaradeva University of Health Sciences, Jaher Pam, 781032 Assam India; 8 Department of Operations, Kasturba Hospital, Manipal Academy of Higher Education, Manipal, 576104 India; 9 Royal Cornwall Hospital Trust, NHS, Truro, TR1 3LJ UK; 10 Department of Cardiovascular and Thoracic Surgery, Kasturba Medical College, Manipal Academy of Higher Education, Manipal, 576104 India

**Keywords:** Cardiometabolic disease model, Menopause and cardiovascular health, Type 2 diabetes mellitus, Experimental cardiovascular research, Translational medicine, Cardiology, Diseases, Endocrinology, Medical research, Physiology

## Abstract

Cardiovascular disease (CVD) remains the leading cause of death in postmenopausal women, often exacerbated by coexisting metabolic disorders such as obesity and type 2 diabetes mellitus. Existing preclinical models fail to capture the multifactorial nature of these overlapping risk factors in a sex-specific context. Here, we present a novel, translationally relevant cardiometabolic model in female Wistar rats that integrates estrogen deficiency, dietary excess, and diabetic stress to mimic postmenopausal disease progression. The model exhibits pronounced cardiometabolic dysfunction, including obesity, insulin resistance, dyslipidemia, QTc prolongation, reduced QRS amplitude, and elevated markers of myocardial injury (Troponin T, CK-MB) and systemic inflammation (IL-6, IL-1β, TNF-α) (*p* < 0.05). These hallmarks closely mirror human postmenopausal cardiometabolic syndrome, providing a clinically relevant platform for mechanistic studies. Notably, this model allows investigation of mitochondrial dysfunction, oxidative stress, and endothelial impairment, while enabling preclinical evaluation of targeted pharmacological interventions, including hormone replacement therapy, metabolic modulators, anti-inflammatory agents, and cardioprotective strategies. By bridging preclinical findings with clinical applications, this approach offers a powerful tool to advance personalized therapeutic strategies for high-risk postmenopausal women, addressing a critical gap in translational cardiovascular research.

## Introduction

Cardiovascular diseases (CVDs) remain the leading cause of mortality among women worldwide, accounting for nearly one-third of all female deaths annually^1^. Despite significant advancements in cardiovascular research, women continue to experience disproportionately high morbidity and mortality from CVDs due to the interplay of traditional and sex-specific risk factors. Conventional risk factors such as diabetes, hypertension, dyslipidemia, obesity, and smoking contribute to cardiovascular risk in both men and women; however, the relative impact of these factors varies significantly based on biological sex. In addition, female-specific risk factors including age at menarche, pregnancy-related complications, polycystic ovary syndrome (PCOS), early or premature menopause, and the use of assisted reproductive technologies exacerbate cardiovascular risk across a woman’s lifespan^2^.

One of the most critical transitions in a woman’s life is menopause, a period marked by a sharp decline in estrogen levels. Estrogen plays a pivotal role in cardiovascular protection, exerting favorable effects on lipid metabolism, vascular function, glucose homeostasis, and inflammatory responses^3^. The loss of estrogen during menopause has been strongly linked to an increased risk of ischemic heart disease (IHD), as it promotes adverse changes in body composition, lipid profiles, and vascular health^4^. Women experiencing premature (< 40 years) or early (< 45 years) menopause face an even greater cardiovascular burden, with an elevated risk of developing heart failure and atrial fibrillation compared to those who undergo menopause at a normal age^5^. The cessation of estrogen’s cardioprotective effects contributes to a cluster of metabolic disturbances, including increased visceral adiposity, insulin resistance, atherogenic dyslipidemia, and hypertension^6^. It has been demonstrated that experimental estrogen depletion via ovariectomy significantly impairs cardiovascular function, further supporting the critical role of estrogen in maintaining cardiac health^7^.

Hormone replacement therapy (HRT) has been considered a possible approach to mitigate the negative cardiovascular effects of estrogen depletion^8,9^. Despite research suggesting improvements in vascular function and lipid metabolism, clinical use of HRT is associated with elevated risks of coronary artery disease, thrombosis, stroke, and pulmonary embolism^10–12^. These limitations emphasize the need for safer, alternative cardioprotective therapies in postmenopausal women.

Menopause is a crucial time for the development and progression of obesity-related problems since it is also strongly linked to increased adiposity and metabolic dysregulation^13^. Menopause-related hormonal changes lead to changes in lipid and glucose metabolism, which in turn cause changes in dietary preferences, preferential storage of abdominal fat, and increased appetite^14^. The resultant increase in visceral fat deposition is a key driver of insulin resistance, type 2 diabetes mellitus (T2DM), hypertension, and dyslipidemia—factors that collectively augment cardiovascular risk^15^.

The pharmacological management of obesity includes the use of Food and Drug Administration (FDA)-approved anti-obesity agents such as sibutramine, orlistat, phentermine, and diethylpropion. These agents are typically reserved for individuals who fail to achieve adequate weight loss through lifestyle modifications alone. However, each of these drugs presents specific challenges. Sibutramine, for example, has been associated with increased blood pressure despite its favorable effects on high-density lipoprotein (HDL) levels and triglycerides. Orlistat, on the other hand, is known to lower low-density lipoprotein (LDL) cholesterol and blood pressure, but its use is often limited by gastrointestinal side effects. Meanwhile, phentermine and diethylpropion, both amphetamine derivatives, carry risks of nausea, dizziness, insomnia, and depression. The search for safer and more effective therapies is particularly critical in postmenopausal women who often present with obesity as part of a broader cardiometabolic syndrome.

Visceral adiposity and insulin resistance are central to the pathophysiology of metabolic syndrome, which significantly increases the risk of CVD^16^. In response to insulin resistance, pancreatic beta cells increase insulin secretion, resulting in compensatory hyperinsulinemia. While this adaptation initially preserves normal glucose levels, it eventually fails, leading to the onset of T2DM^17^. The chronic hyperglycemia characteristic of T2DM accelerates atherosclerosis, predisposing individuals to coronary artery disease, cerebrovascular disease, and peripheral artery disease^18^.

The management of T2DM primarily involves lifestyle modifications, including dietary adjustments, regular physical activity, and weight reduction. However, when these measures are insufficient, pharmacologic intervention becomes necessary^19–21^. Oral hypoglycemic agents such as sulfonylureas are widely used but pose risks of hypoglycemia and weight gain. Alpha-glucosidase inhibitors, while effective in controlling postprandial glucose levels, frequently cause gastrointestinal side effects such as bloating and diarrhea. Insulin therapy remains a cornerstone of diabetes management but is associated with weight gain and an increased risk of hypoglycemic episodes. Given the high burden of diabetes-related cardiovascular complications, there is an urgent need for therapeutic approaches that address both glycemic control and cardiovascular protection.

The complex interrelationship between menopause, obesity, and T2DM contributes synergistically to the development of ischemic heart disease. Despite the availability of pharmacologic therapies, these conditions are often treated independently, potentially undermining overall cardiovascular outcomes. Additionally, treatment-related adverse effects compromise patient adherence and quality of life while increasing healthcare costs^22–24^. Thus, developing safer, multifaceted, and targeted therapies is essential to reduce cardiovascular risk in postmenopausal women.

A major barrier in advancing this research is the lack of an integrated preclinical model that captures the interplay between estrogen deficiency, obesity, and diabetes in the context of cardiovascular disease. While individual models for these conditions exist, few reproduce their combined effects, limiting translational relevance^25,26^.

To our knowledge, no existing preclinical model simultaneously incorporates postmenopausal estrogen deficiency, high-fat diet-induced obesity, and chemically induced type 2 diabetes to simulate the multifactorial cardiovascular risk seen in women. This represents a critical gap in preclinical cardiovascular research.

To address this gap, we developed a comprehensive preclinical model in female Wistar rats combining bilateral ovariectomy, high-fat diet-induced obesity, and low-dose streptozotocin-induced diabetes. This model aims to mimic postmenopausal physiology and related metabolic alterations to provide a translational platform for mechanistic studies and therapeutic evaluations. It further allows investigation of the pathological and temporal interactions among hormonal and metabolic changes contributing to ischemic heart disease, offering insights with potential clinical applications^27,28^.

## Materials and methods

### Drugs and reagents

The assay kits and essential chemical agents used in this investigation were purchased from reliable vendors to guarantee the precision and dependability of the findings. Nicotinamide (NA) (# N0636) and streptozotocin (STZ) (# S0130), which are necessary for the induction of diabetes, were acquired from Sigma-Aldrich-Merck Ltd. in Bangalore, India. Coral Clinical Systems Ltd., Goa, India, provided the triglycerides (TG) (# 1102230075), total cholesterol (TC) (# 1102042150), and high-density lipoprotein cholesterol (HDL-C) (# 1102150040) and creatine kinase-MB (CK-MB) (# 1102070210) assay kits required to evaluate metabolic and cardiac parameters. Furthermore, Elabscience, USA, provided the enzyme-linked immunosorbent assay (ELISA) kits needed to quantify important biomarkers such as insulin (# E-EL-R3034), Troponin T (# E-EL-R0151), interleukin-6 (IL-6) (# E-EL-R0015), interleukin-1 beta (IL-1β) (# E-EL-R0012), and tumor necrosis factor-alpha (TNF-α) (# E-EL-R2856). To preserve these reagents’ stability and effectiveness over the study period, they were handled and kept in compliance with the manufacturer’s instructions.

### Experimental animals

A total of 12 female Wistar rats (150–200 g; 8–10 weeks old) used in this investigation were acquired from an approved animal breeding facility- Central Animal House Facility, Manipal Academy of Higher Education, Manipal, India. Identical housing conditions were maintained across all groups, and environmental enrichment measures were applied uniformly. This ensures that any residual influence of individual housing was systematically controlled, preserving the internal validity and translational reliability of our findings^29–32^. The rats were fed a typical rat pellet diet procured from a local food supplier and had unlimited access to tap water. The animals were given a week to acclimate to the laboratory environment before the experimental procedures started^33–35^. Fasting protocols involved a 10-hour food deprivation period, during which access to water remained unrestricted^36–38^. The sample size was determined to be 12 (six animals per group) using the standard G*Power formula, with Z_α/2_ set at 1.96 to ensure a 95% confidence level and Z_β_ set at 0.84 to achieve 80% statistical power. This sample size is in accordance with the ethical principle of Reduction of number of animals. This study fully adhered to the ethical principles of the 3Rs (Replacement, Reduction, Refinement) to ensure the humane treatment and well-being of the animals involved. All experimental protocols were conducted in strict adherence to ethical guidelines and were reviewed and approved by the Institutional Animal Ethics Committee (IAEC/KMC/107/2021). The study complies with the ethical standards outlined by the Committee for Control and Supervision of Experiments on Animals (CCSEA), Government of India, ensuring the welfare and humane treatment of the animals. Furthermore, this study aligns with the ARRIVE (Animal Research: Reporting In Vivo Experiments) guidelines, ensuring transparency, reproducibility, and ethical rigor in animal research, with a focus on minimizing animal distress and maximizing scientific value.

### Experimental design

**Fig. 1:**
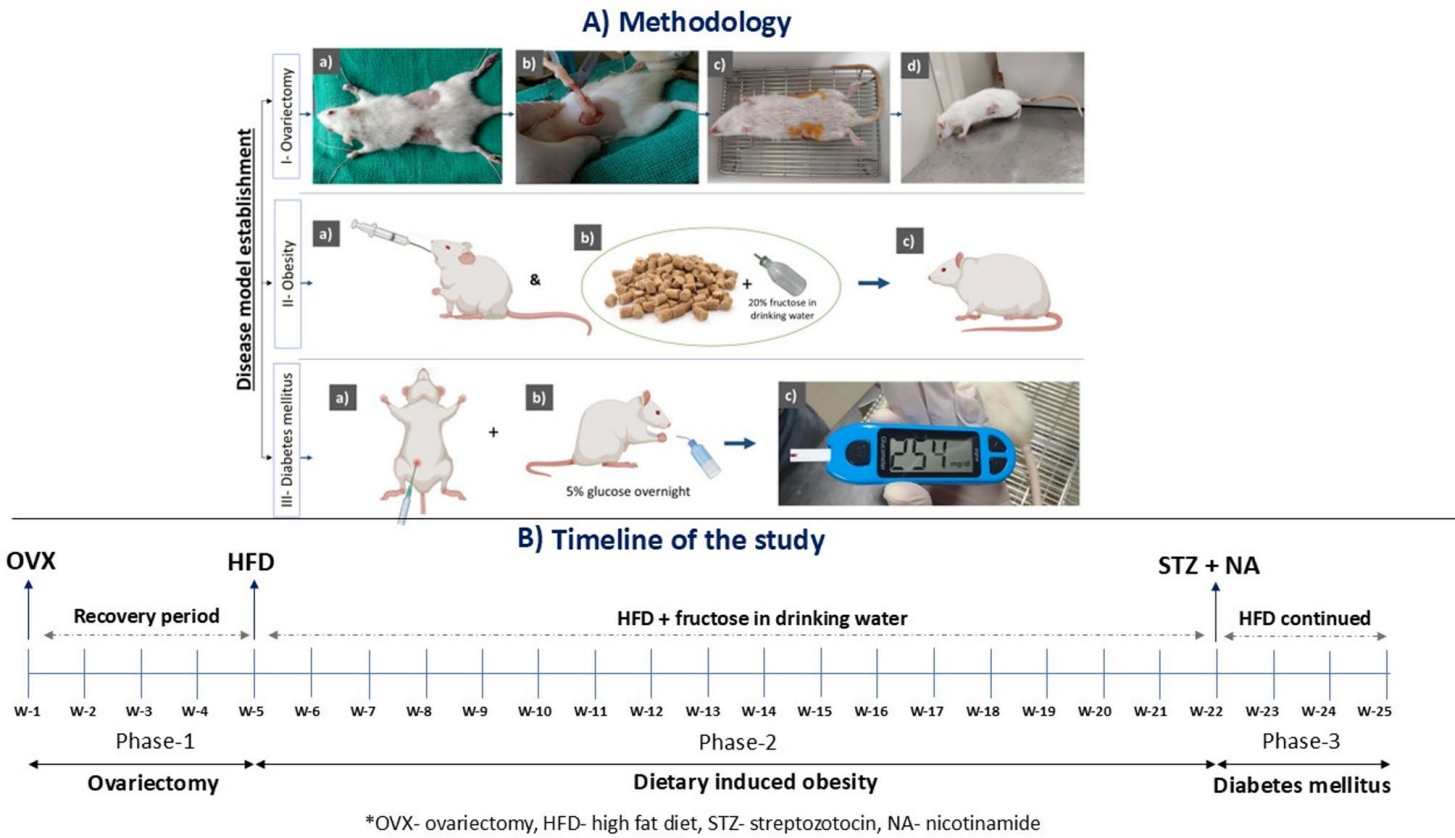
Schematic representation of the experimental design for inducing ovariectomy, obesity, and diabetes mellitus in rats. (**A**) Methodology: Depicts the procedural steps for establishing each disease model. I-Ovariectomy (a–d) illustrates surgical procedures on a rat, including shaving, positioning, bilateral ovariectomy, and post-operative recovery. II-Obesity (a-c) shows induction via high-fat diet (vanaspati dalda + coconut oil) and fructose in drinking water, leading to body weight exceeding 300 g. III-Diabetes mellitus (a-c) demonstrates intraperitoneal administration of nicotinamide and streptozotocin, followed by glucose supplementation, and confirmation via fasting blood glucose levels above 200 mg/dL. (**B**) Timeline of the study: Presents the 25-week study timeline, outlining the phases for ovariectomy (OVX), high-fat diet (HFD) induction, and diabetes mellitus (DM) development. The stepwise experimental design emulates real-world human pathophysiology: Phase I (OVX) models post-menopausal estrogen deficiency, which predisposes women to cardiometabolic disorders; Phase II (HFD) reflects diet-induced obesity as seen in sedentary lifestyles; and Phase III (nicotinamide-STZ) simulates adult-onset insulin-resistant type 2 diabetes. This sequential model provides a highly relevant preclinical framework for testing cardiometabolic interventions in females.

The experimental rats were assigned to two groups (*n* = 6 per group) using a simple randomization method, wherein each animal was allocated a unique identification number, and group assignment was determined using a computer-generated random number table to ensure unbiased distribution and minimize selection bias. This randomization approach is consistent with the ARRIVE guidelines for in vivo animal research. Group I - Control group and Group II - Disease group. The control group underwent a sham operation to serve as a control for ovariectomy, was fed a standard rat pellet diet to counteract as control for high-fat diet-induced obesity, and received normal saline (1 mL/kg, intraperitoneally), followed by an intraperitoneal injection of 0.1 M cold sodium citrate buffer (pH 4.5) to mimic the vehicle control conditions for nicotinamide and streptozotocin administration used in diabetes mellitus induction in the disease group. The total duration of this study was 25 weeks, which was carefully chosen to align with the physiological timeline of disease progression in humans. In adult rats, each day is approximately equivalent to 34.8 human days, meaning that one rat month corresponds to nearly three human years^39^. Based on this conversion, the 25-week experimental period in rats translates to approximately 6,090 human days or ~ 16 years and 8 months. This extended duration was designed to mimic chronic disease development and progression in humans, ensuring the reliability and translational value of the study. To achieve this, the disease group rats underwent a structured three-phase experimental protocol aimed at establishing a robust and reproducible preclinical multifactorial cardiometabolic disease model (Fig. 1).

#### Phase I: ovariectomy (OVX) model establishment

In Week 1, ovariectomy was performed on disease group rats following the anesthesia which was induced by intraperitoneal administration of ketamine (60 mg/kg) and xylazine (10 mg/kg). The anesthetized rats were positioned in a ventral recumbency posture. The surgical site was shaved and disinfected using povidone-iodine solution. A dorso-lateral incision was made between the caudal ribcage and the base of the tail, exposing the ovary and oviduct through the abdominal muscle wall incision.

The oviduct was ligated using a sterile 3 − 0 (3 metric), 20 mm chromic absorbable surgical suture (NW 4237; Ethicon, USA), and the ovary together with a portion of the oviduct was excised in a single cut. The remaining tissue was repositioned within the peritoneal cavity before suturing the incision. The contralateral ovary was removed following the same procedure^40^. Postoperative care included intraperitoneal administration of 1 mL of 0.9% normal saline on both the dorso-lateral surgical sides on the day of surgery, and wound management was performed everyday using topical povidone-iodine until complete healing was achieved.

#### Phase II: high-fat diet (HFD)-induced obesity

Four weeks after ovariectomy, the disease group was placed on a high-fat diet (HFD) from Week 5 to Week 25 (a total duration of 21 weeks). The HFD consisted of a 3:1 mixture of vanaspati dalda (sourced from Ruchi Soya Industries Ltd., Indore, India) and coconut oil (obtained from KLF Nirmal Industries Pvt. Ltd., Thrissur, India) administered orally at 10 mL/kg/day, along with a standard rat pellet diet^41,42^. The detailed caloric and nutrient composition of all diet (both normal and high fat diet) components used in this study has been provided in Table 1. Additionally, 20% (4 kcal/g; total 80 kcal) fructose was supplemented in the drinking water throughout week 5 to week 25 like HFD feeding. Body weight was measured weekly, and rats reaching a body weight of ≥ 300 g were classified as obese and subsequently progressed to the next phase. The high-fat diet included mixture of vanaspati dalda and coconut oil in 3:1 ratio, chosen for its ability to mimic human dietary saturated fat intake associated with visceral adiposity and insulin resistance. The concurrent administration of 20% fructose in drinking water was indented to potentiate hepatic lipogenesis and elevate triglyceride levels, facilitating a rapid onset of dyslipidemia and insulin resistance, thereby enhancing the translational value of the model.

**Table 1:** Caloric and nutrient composition of the standard rat pellet diet (normal diet) and the high-fat diet used in the experimental protocol.

Caloric and nutrient composition	Standard rat pellet diet (normal diet)	High-fat diet (mixture of vanaspati dalda and coconut oil in 3:1 ratio; 10 mL/kg/day, *p*.o.)	
		Vanaspati Dalda	Coconut Oil
Energy	3.0–3.5 kcal/g	899.64 kcal/100 g	900 kcal/100 g
Crude protein	20%	–	–
Crude fat	5%	–	–
Crude fiber	5%	–	–
Minerals	8%	–	–
Carbohydrates	60%	–	–
Total fat	–	99.96 g	99.90 g
Saturated fat	–	≤ 63.03 g	91.7 g
Trans fat	–	≤ 2 g	–
Monounsaturated fat	–	30.09 g	6.6 g
Polyunsaturated fat	–	6.84 g	1.6 g
Omega-6 fatty acids	–	–	1.6 g

The standard rat pellet diet served as the normal control diet, while the high-fat diet consisted of a mixture of vanaspati dalda and coconut oil in a 3:1 ratio, administered at 10 mL/kg/day by oral gavage (p.o.). Energy and macronutrient compositions are expressed per gram or 100 g of diet as appropriate.

*kcal* kilocalorie, *g* gram, *p.o.* per os (oral administration).

#### Phase III: diabetes mellitus induction

In Week 22, during the progression of high-fat diet-induced obesity in the disease group, diabetes mellitus was induced through an intraperitoneal injection of nicotinamide (110 mg/kg), followed by streptozotocin (50 mg/kg) dissolved in 0.1 M cold sodium citrate buffer (pH 4.5) and kept on ice until administration^43–46^. The animals were allowed to drink 5% glucose solution overnight to overcome the drug-induced hypoglycemia. One week after injection (Week 23), rats with fasting blood glucose levels of ≥ 200 mg/dL were classified as diabetic^47^. The high-fat diet was continued until Week 25 for the disease group.

### Body weight measurement

Body weight of all animals was measured weekly throughout the experimental phase using a calibrated digital weighing balance. Measurements were consistently performed in the morning after overnight fasting, ensuring standardization and minimizing variability.

### Electrocardiographic (ECG) recording

After 25 weeks of experimental intervention, ECG recordings were performed under anesthesia (induced via intraperitoneal injection of ketamine 60 mg/kg and xylazine 10 mg/kg) for all animals while they were positioned on an operating table. Electrodes were attached to the palmar surfaces of the front limbs and the left hind limb, while the right hind limb was connected to a grounded electrode. A conductive ECG gel was applied to ensure optimal signal acquisition and to prevent electrical interference.

Using a BPL Cardiart 9108 ECG machine, standard lead II ECG tracings were recorded for one minute per animal. The obtained ECG signals were analyzed both qualitatively and quantitatively, with validation by an interventional cardiologist. The following parameters were assessed: PR interval, QT interval, corrected QT (QTc) interval, and QRS-complex amplitude^48,49^. The QTc interval was calculated using Bazett’s formula (QTc = QT / √RR)^50^.

### Biochemical analysis

#### Fasting blood glucose measurement

Following ECG recording, fasting blood glucose levels were measured in all animals using the glucose oxidase–peroxidase reactive strip-based method with an Accu-Chek glucometer (Roche Diagnostics, USA).

#### Blood collection and serum Preparation

While under the same anesthetic effect following ECG recording and fasting blood glucose measurement, 2 mL of blood was collected from the retro-orbital venous plexus using capillary tubes. A Remi C-24 refrigerated centrifuge was used to centrifuge the collected blood at 3000 rpm for 20 min at 4 °C after it had been stored in microcentrifuge tubes and allowed to clot. The resultant serum was kept cold (-80 °C) until it could be analyzed further^51,52^.

#### Lipid profile estimation

Serum levels of triglycerides (TG), total cholesterol (TC), and high-density lipoprotein cholesterol (HDL-C) were quantified using a semi-autoanalyzer (Star 21 Plus, Mumbai, India). Very low-density lipoprotein cholesterol (VLDL-C) and low-density lipoprotein cholesterol (LDL-C) was calculated using Friedewald’s equation: VLDL-C = TG/5; LDL-C = TC – (HDL-C + VLDL-C) respectively^53,54^. Atherogenic Risk Index (ARI) and Coronary Risk Index (CRI) was calculated using the formula: ARI = (TC - HDL-C) / HDL-C; CRI = TC / HDL-C respectively.

#### Cardiac biomarker analysis

Following the manufacturer’s instructions, the sandwich-ELISA method was used to measure the amount of troponin T, and a BIO-RAD iMark microplate reader was used to quantify absorbance at 450 nm. The semi-autoanalyzer (Star 21 Plus, Mumbai, India) was used to measure the CK-MB levels.

#### Insulin resistance and insulin sensitivity analysis

The Bio-Rad iMark microplate reader was used to read the optical density for insulin levels, which were determined using respective ELISA kit (Elabscience, USA). Homeostatic model assessment of insulin resistance (HOMA-IR) values provide an integrated measure of insulin sensitivity, critical for evaluating the metabolic impact of estrogen deficiency, obesity, and diabetes.

HOMA-IR was calculated using the formula^55^: HOMA-IR = (Fasting Blood Glucose [mg/dL] x Fasting Insulin [µU/mL]) / 405.

Quantitative insulin-sensitivity check index (QUICKI) values serve as an inverse index of insulin resistance and a reliable surrogate marker of insulin sensitivity, providing critical insight into the metabolic alterations associated with estrogen deficiency, obesity, and diabetes.

The formula for the calculation of QUICKI is as follows: QUICKI = 1/[log(I_o_) + log(G_o_)] Where: I_o_ is fasting insulin (µU/mL) and G_o_ is fasting glucose (mg/dL)^56^.

#### Estimation of IL-6, IL- 1β and TNF α

Serum was used to measure inflammatory cytokines to assess systemic inflammation in our model of postmenopausal cardiometabolic risk. This approach captures the combined effects of hormonal deficiency and dietary intervention, on circulating cytokines, providing relevant translational insight while avoiding additional tissue processing. The quantification of IL-6, IL-1β and TNF α levels were performed using the Sandwich-ELISA method, following standard protocols provided with the respective ELISA kits. The absorbance measurements were taken at 450 nm using a microplate reader (BIO-RAD iMarkTM microplate reader, USA).

### Heart isolation, gross examination, and histopathological analysis

Following blood collection, euthanasia was induced by administering intraperitonially an additional dose of ketamine, bringing the total dosage to approximately 120 mg/kg, in accordance with the Committee for Control and Supervision Experiments on Animals (CCSEA) guidelines, Government of India (Annexure-6: Euthanasia of Laboratory Animals). A surgical scalpel was used to open the thoracic cavity, and the heart was carefully excised from the mediastinum by dissecting it from the major blood vessels. After gross morphological examination, the heart was fixed in 10% formalin for histopathological analysis.

The tissue was fixed in 10% formalin for 24 h, then dehydrated sequentially in 50% ethanol (48 h), 70% ethanol (24 h), 90% ethanol (24 h), and 100% ethanol (24 h). The samples were then cleared in xylene until translucent. Paraffin embedding was performed using embedding cassettes, and the blocks were stored at 18 °C for 24 h.

Histological sections of 5 μm thickness were cut using a rotary microtome. The sections were floated in a water bath and mounted on lysine-coated slides. After drying on a hot plate, the slides were stained using Hematoxylin and Eosin (H&E) for general tissue architecture and Masson’s Trichrome (MT) for evaluation of fibrosis and collagen deposition^49^.

### Statistical analysis

All experimental data were analyzed using SPSS Version 29.0. Data are presented as mean ± standard deviation (SD). The normality of each variable was assessed using the Shapiro-Wilk test, and the majority of variables showed non-significant p-values (*p* > 0.05), supporting the assumption of normality. Comparisons between the two groups were performed using an independent-samples t-test. In addition to p-values, effect sizes (Cohen’s d) and 95% confidence intervals (CI) were calculated for all variables to provide a measure of both the magnitude and precision of the observed differences. Statistical significance was defined as *p* < 0.05.

## Results

### Effect on fasting blood glucose level and body weight

The progression of metabolic alterations in the disease group was evident through significant changes in fasting blood glucose levels and body weight over time. In comparison to the control group, the disease group animals exhibited a substantial rise in fasting blood glucose (*p* < 0.001), indicating the onset of metabolic dysfunction. This rise emphasizes how metabolic disorders and ovariectomy affect glucose control.

The body weight of disease group animals following ovariectomy exhibited a noticeable rising trend in tandem with the increase in blood glucose levels. Rapid metabolic changes brought on by the experimental settings were highlighted by the fact that by the fifth week, the disease group’s body weight had significantly increased (*p* = 0.002) in comparison to the control group. The disease group was further distinguished from the control group animals over the experimental period by a continuous and statistically significant rise (*p* < 0.001) that was seen from this point on.

**Fig. 2:**
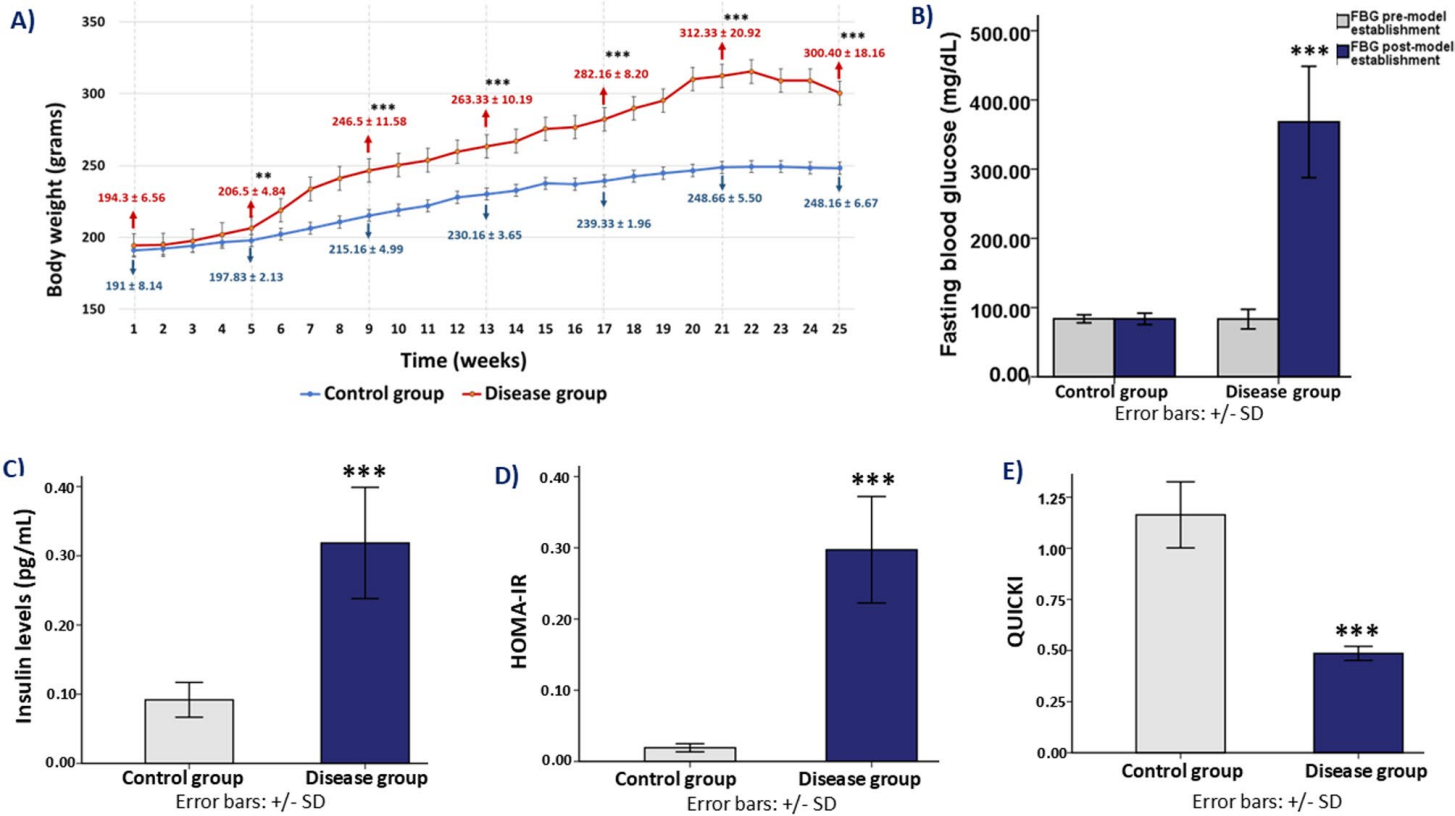
Physiological changes in rats following induction of ovariectomy, obesity, and diabetes mellitus. (**A**) Body weight changes: Graph depicting the change in body weight (grams) over 25 weeks in both control group and disease group. (**B**) Fasting blood glucose (FBG): Bar graph comparing FBG levels (mg/dL) pre- and post-model establishment in both groups. (**C**) Insulin levels: Bar graph comparing insulin levels (pg/mL) between control group and disease group. (**D**) HOMA-IR: Bar graph comparing Homeostatic Model Assessment for Insulin Resistance (HOMA-IR) between control group and disease group. (**E**) QUICKI: Bar graph comparing Quantitative Insulin Sensitivity Check Index (QUICKI) between the control group and disease group. *** indicates statistical significance (*p* < 0.001) between the control group and disease group.

There was no statistically significant difference in the mean body weights of the two groups at baseline, with the control group weighing an average of 191 ± 8.14 g and the disease group weighing an average of 194.33 ± 6.56 g (*p* = 0.453). In contrast to the control group, which maintained its weight at 248.16 ± 6.67 g, the disease group showed a significant rise in body weight by the end of the research, averaging 300.40 ± 18.16 g (*p* < 0.001) (Fig. 2). This dramatic difference further underscores the metabolic disturbances associated with the disease condition.

### Effect on insulin, HOMA-IR and QUICKI

Insulin resistance was significantly increased in the disease group, as evidenced by elevated insulin levels and homeostatic model assessment of insulin resistance (HOMA-IR) values. Insulin levels were significantly higher in the disease group (0.31 ± 0.08 pg/mL) compared to the control group (0.09 ± 0.02 pg/mL, *p* < 0.001). In the same way, the disease group’s HOMA-IR value (0.29 ± 0.07) was significantly higher than those of the control group (0.01 ± 0.005, *p* < 0.001). In line with these findings, the quantitative insulin sensitivity check index (QUICKI), a validated surrogate marker inversely correlated with insulin resistance, was significantly reduced in the disease group (0.48 ± 0.03) compared to the control group (1.16 ± 0.16, *p* < 0.001), further confirming the presence of impaired insulin sensitivity (Fig. 2).

### Effect on lipid profile

Significant changes in cardiovascular risk indicators and lipid profiles were noted in the disease group, which also showed profound dyslipidemia. The disease group had significantly lower levels of HDL-C (*p* = 0.002) and significantly higher levels of triglycerides, total cholesterol, and low-density lipoprotein cholesterol (LDL-C) (*p* < 0.001), which further supported the atherogenic risk linked to metabolic dysfunction.

In particular, the disease group had considerably higher total cholesterol levels (184.34 ± 21.52 mg/dL) than the control group (77.67 ± 8.78 mg/dL, *p* < 0.001). Additionally, the disease group’s triglyceride levels were significantly higher (292.15 ± 20.62 mg/dL) than those of the control group (169.50 ± 11.87 mg/dL, *p* < 0.001). In contrast to the control group (29.47 ± 6.59 mg/dL, *p* = 0.002), the HDL-C levels in the disease group decreased significantly (16.05 ± 4.44 mg/dL).

Furthermore, the LDL-C levels of the disease group were substantially higher (109.86 ± 16.06 mg/dL) than those of the control group (14.30 ± 5.03 mg/dL, *p* < 0.001). Likewise, the very-low-density lipoprotein cholesterol (VLDL-C) levels in the disease group were substantially higher (58.43 ± 4.12 mg/dL) than in the control group (33.90 ± 2.37 mg/dL, *p* < 0.001).

**Fig. 3:**
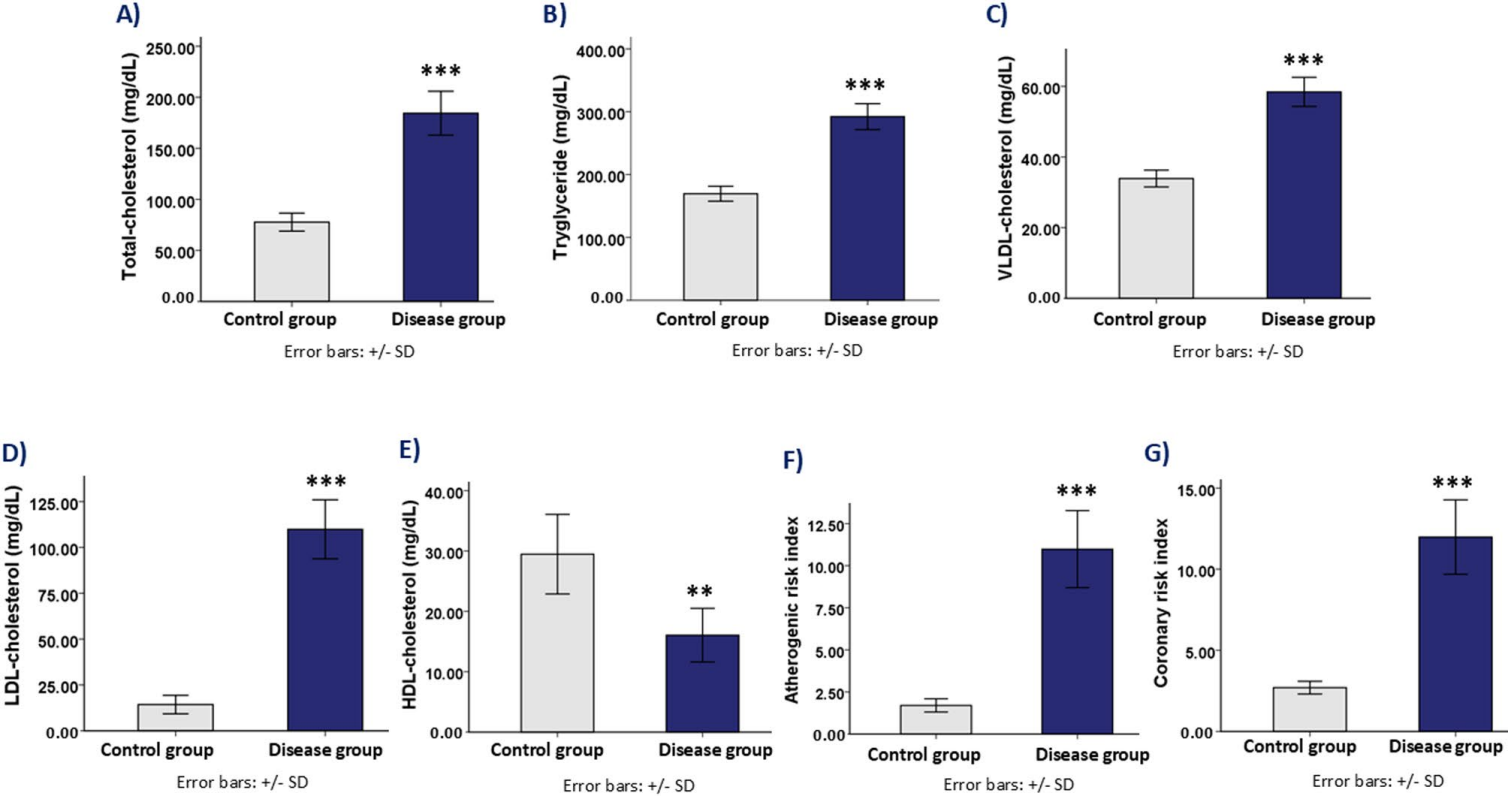
Lipid profile and cardiovascular risk assessment in control group and disease group rats. (**A**) Total Cholesterol: Bar graph comparing total cholesterol levels (mg/dL) between control group and disease group. (**B**) Triglyceride: Bar graph comparing triglyceride levels (mg/dL) between control group and disease group. (**C**) VLDL-Cholesterol: Bar graph comparing very low-density lipoprotein cholesterol (VLDL-cholesterol) levels (mg/dL) between control group and disease group. (**D**) LDL-Cholesterol: Bar graph comparing low-density lipoprotein cholesterol (LDL-cholesterol) levels (mg/dL) between control group and disease group. (**E**) HDL-Cholesterol: Bar graph comparing high-density lipoprotein cholesterol (HDL-cholesterol) levels (mg/dL) between control group and disease group. (**F**) Atherogenic Risk Index (ARI): Bar graph comparing the atherogenic risk index between control group and disease group. (**G**) Coronary Risk Index (CRI): Bar graph comparing the coronary risk index between control group and disease group. **Note**: *** indicates statistical significance (*p* < 0.001) between the control group and disease group.

The disease group had significantly higher atherogenic risk index (ARI) and cardiac risk index (CRI) values (ARI: 10.98 ± 2.29; CRI: 11.98 ± 2.29) than the control group (ARI: 1.69 ± 0.39; CRI: 2.69 ± 0.39, *p* < 0.001 for both) (Fig. 3). This demonstrates that metabolic dysfunction is linked to an increased risk of cardiovascular disease.

### Effect on ECG

Electrophysiological disturbances in cardiac function were prominent in the disease group, as reflected in the electrocardiogram (ECG) findings. A depressed ST segment, lack or inversion of the P wave, nonspecific ST segment alterations, or ST segment elevation were among the anomalies that some animals displayed during the initial screening. To guarantee a consistent baseline for further analysis, these animals were removed from the experiment and swapped out for healthy rats.

Significant differences in ECG parameters between the disease group and the control group were observed as the experiment went on. Significantly longer PR intervals (70.00 ± 10.95 ms) were seen in disease group animals compared to control groups (45.00 ± 8.36 ms), with a highly significant p-value of 0.001. In addition, the QRS complex amplitude was markedly reduced in disease group animals (146.66 ± 20.65 ms) compared to control groups (200.00 ± 25.29 ms), with a p-value of 0.003, indicating potential myocardial electrical disturbances.

Furthermore, with a p-value of 0.001, the QT interval was considerably longer in the disease group (88.33 ± 9.83 ms) than in the control group (60.00 ± 12.64 ms). Similarly, the QTc interval exhibited a significant increase in disease group animals (168.33 ± 20.41 ms) relative to control group (93.33 ± 16.32 ms), with a p-value of < 0.001 (Fig. 3). These alterations suggest compromised cardiac electrophysiology, possibly predisposing the disease group animals to arrhythmic events.

**Fig. 4:**
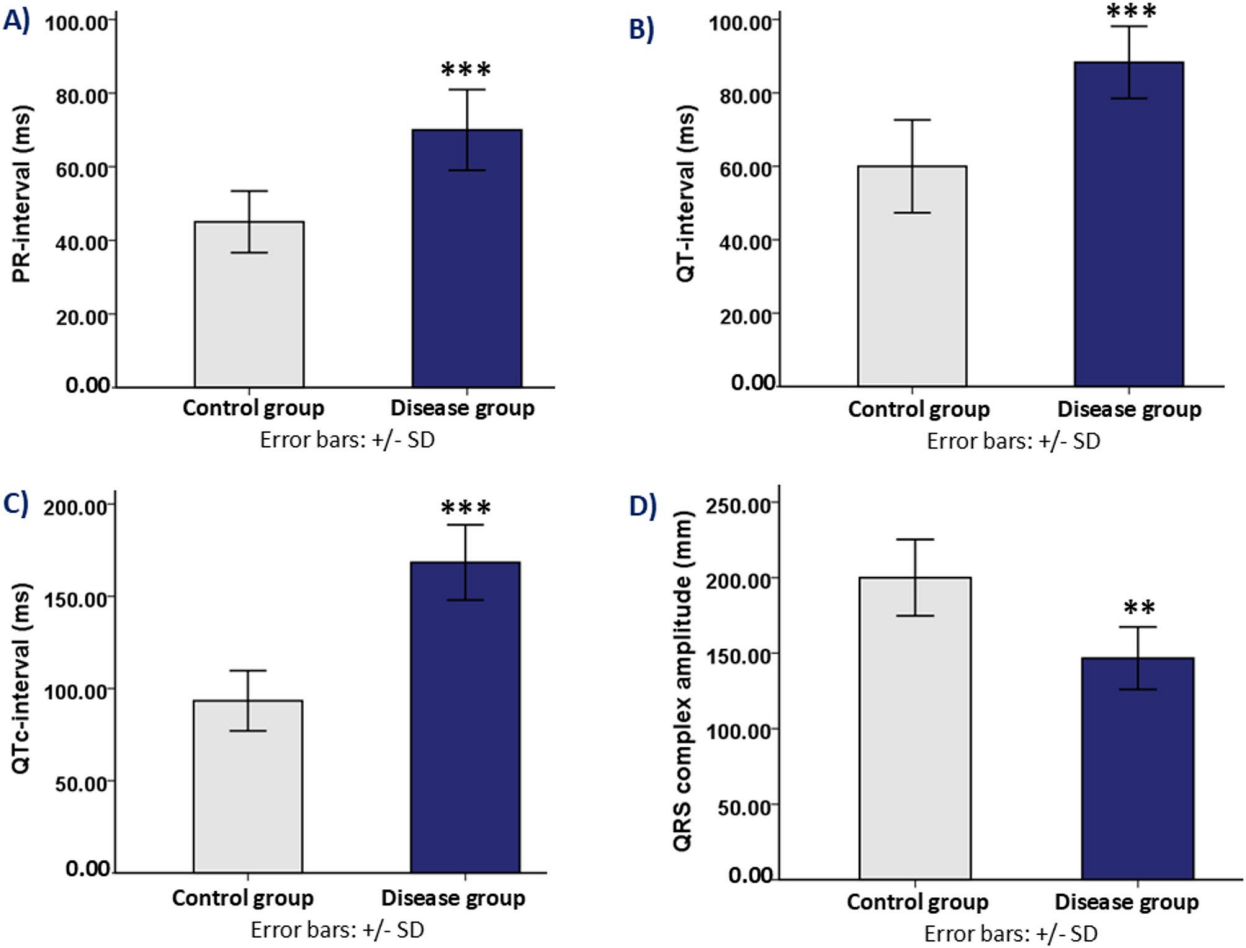
Electrocardiographic recording in control group and disease group rats. (**A**) PR-interval: Bar graph showing the PR-interval (ms) in both control group and disease group. (**B**) QT-interval: Bar graph showing the QT-interval (ms) in both control group and disease group. (**C**) QTc-interval: Bar graph showing the corrected QT-interval (QTc, ms) in both control group and disease group. (**D**) QRS complex amplitude: Bar graph showing the amplitude of the QRS complex (mm) in both control group and disease group. Note: *** indicates statistical significance (*p* < 0.001) between the control group and disease group. ** indicates statistical significance (*p* < 0.01) between the control group and disease group.

### Effect on cardiac injury biomarkers (CK-MB and troponin T)

**Fig. 5:**
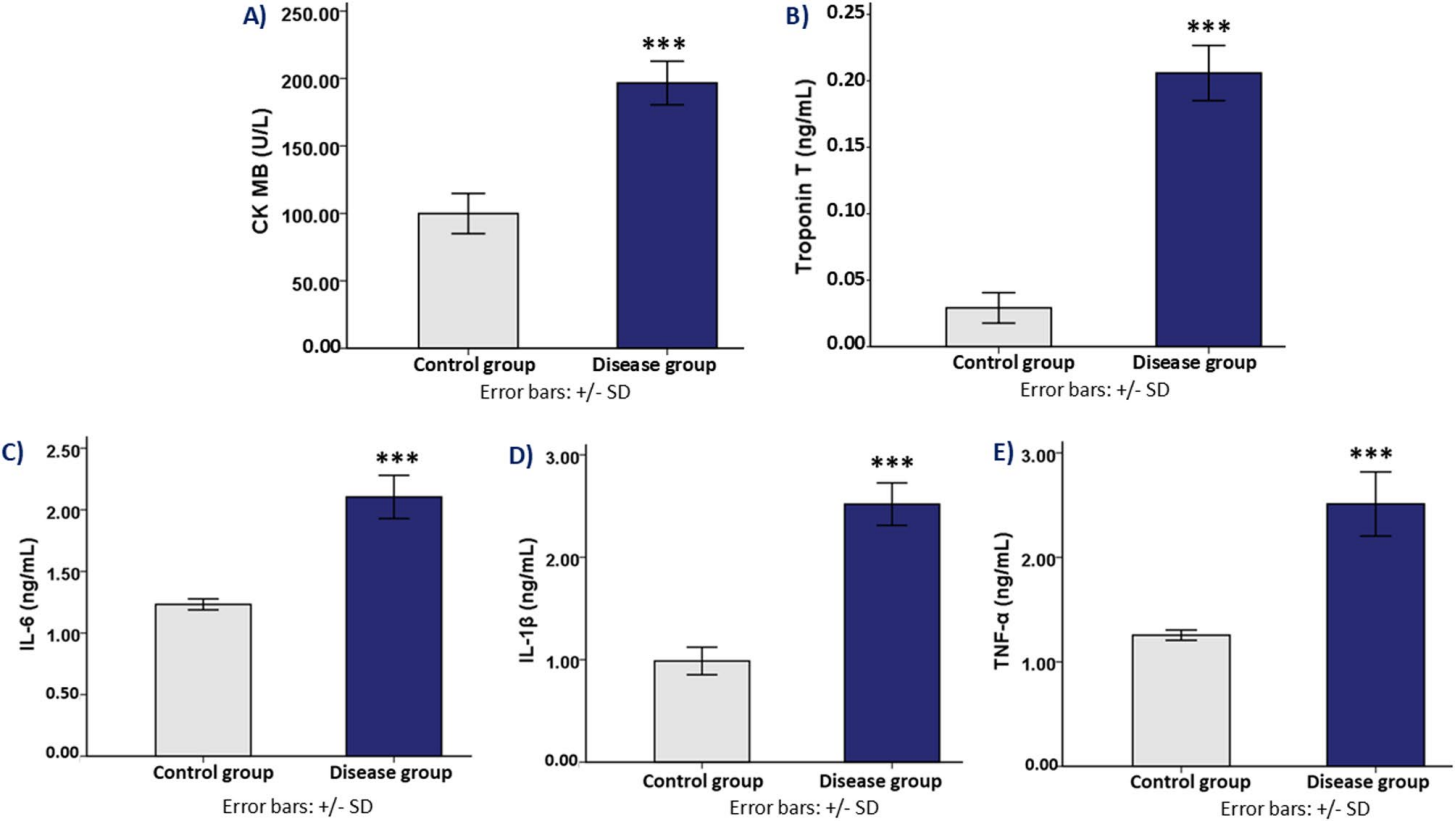
Cardiac injury biomarkers and inflammatory cytokine levels in control group and disease group rats. (**A**) CK-MB: Bar graph showing the creatine kinase-MB (CK-MB) levels (U/L) in both control group and disease group. (**B**) Troponin T: Bar graph showing the troponin T levels (ng/mL) in both control group and disease group. (**C**) IL-6: Bar graph comparing interleukin-6 (IL-6) levels (ng/mL) between control group and disease group. (**D**) IL-1β: Bar graph comparing interleukin-1 beta (IL-1β) levels (ng/mL) between control group and disease group. (**E**) TNF-α: Bar graph comparing tumor necrosis factor alpha (TNF-α) levels (ng/mL) between control group and disease group. *** indicates statistical significance (*p* < 0.001) between the control group and disease group. The statistical comparison of quantitative outcome variables between experimental groups is summarized in Table 2.

Markers of cardiac injury were significantly elevated in the disease group, suggesting ongoing myocardial damage. CK-MB levels were significantly elevated in the disease group (196.64 ± 16.19 U/L) relative to the control group (99.87 ± 14.89 U/L, *p* < 0.001). Similarly, Troponin T levels were markedly increased in the disease group (0.20 ± 0.02 ng/mL) compared to the control group (0.02 ± 0.01 ng/mL, *p* < 0.001). (Fig. 5- A & B). These findings indicate potential myocardial stress or injury in the disease group animals.

### Effect on inflammatory cytokines (IL-6, IL-1β, and TNF-α)

Inflammatory cytokine levels were significantly higher in the disease group, suggesting a pro-inflammatory condition. The disease group had higher levels of IL-6 (2.10 ± 0.17 ng/mL) than the control group (1.23 ± 0.04 ng/mL, *p* < 0.001). Similarly, IL-1β levels in the disease group were significantly higher (2.51 ± 0.20 ng/mL) than in the control group (0.98 ± 0.13 ng/mL, *p* < 0.001). In comparison to the control group (1.25 ± 0.04 ng/mL), TNF-α levels also increased significantly in disease group (2.51 ± 0.30 ng/mL, *p* < 0.001) (Fig. 5- C, D & E).

**Table 2:** Statistical summary of quantitative outcome variables among experimental groups.

Outcome variables	Groups (*n* = 6/group)	Mean	Standard deviation (±)	95% confidence interval	Mean difference	Cohen’s d	*p* value
FBG (mg/dL)	Control	83.67	8.29	74.95–92.39	284.50	4.97	< 0.001^$^
	Disease	368.17	80.45	283.71–452.63			
Baseline body weight (g)	Control	191.00	8.14	182.45–199.55	3.33	0.45	0.453^#^
	Disease	194.33	6.56	187.44–201.22			
Final body weight (g)	Control	248.16	6.67	241.17–255.15	52.24	3.82	< 0.001^$^
	Disease	300.40	18.16	281.34–319.46			
Insulin (pg/mL)	Control	0.09	0.02	0.069–0.111	0.22	3.79	< 0.001^$^
	Disease	0.31	0.08	0.226–0.394			
HOMA-IR	Control	0.01	0.005	0.0048–0.0153	0.28	5.65	< 0.001^$^
	Disease	0.29	0.07	0.216–0.364			
QUICKI	Control	1.16	0.16	0.992–1.328	0.68	5.91	< 0.001^$^
	Disease	0.48	0.03	0.449–0.511			
TC (mg/dL)	Control	77.67	8.78	68.46–86.88	106.67	6.49	< 0.001^$^
	Disease	184.34	21.52	161.77–206.91			
TG (mg/dL)	Control	169.50	11.87	157.05–181.95	122.65	7.29	< 0.001^$^
	Disease	292.15	20.62	270.51–313.79			
VLDL-C (mg/dL)	Control	33.90	2.37	31.41–36.39	24.53	7.30	< 0.001^$^
	Disease	58.430	4.12	54.11–62.75			
LDL-C (mg/dL)	Control	14.30	5.03	9.03–19.57	95.56	8.03	< 0.001^$^
	Disease	109.86	16.06	92.99–126.73			
HDL-C (mg/dL)	Control	29.47	6.59	22.56–36.38	13.42	2.39	0.002^$^
	Disease	16.05	4.44	11.40–20.70			
ARI	Control	1.69	0.39	1.28–2.10	9.29	5.67	< 0.001^$^
	Disease	10.98	2.29	8.58–13.38			
CRI	Control	2.69	0.39	2.28–3.10	9.29	5.67	< 0.001^$^
	Disease	11.98	2.29	9.58–14.38			
PR interval (ms)	Control	45.00	8.36	36.24–53.76	25.00	2.57	0.001^$^
	Disease	70.00	10.95	58.51–81.49			
QT interval (ms)	Control	60.00	12.64	46.73–73.27	28.33	2.50	0.001^$^
	Disease	88.33	9.83	78.03–98.63			
QTc interval (ms)	Control	93.33	16.32	76.18–110.48	75.00	4.06	< 0.001^$^
	Disease	168.33	20.41	146.92–189.74			
QRS complex (mm)	Control	200.00	25.29	173.47–226.53	53.34	2.31	0.003^$^
	Disease	146.66	20.65	125.00–168.32			
CK-MB (U/L)	Control	99.87	14.89	84.25–115.49	96.77	6.23	< 0.001^$^
	Disease	196.64	16.19	179.67–213.61			
Troponin T (ng/mL)	Control	0.02	0.01	0.0095–0.0305	0.18	11.39	< 0.001^$^
	Disease	0.20	0.02	0.179–0.221			
IL-6 (ng/mL)	Control	1.23	0.04	1.188–1.272	0.87	7.05	< 0.001^$^
	Disease	2.10	0.17	1.922–2.278			
IL-1β (ng/mL)	Control	0.98	0.13	0.844–1.116	1.53	9.07	< 0.001^$^
	Disease	2.51	0.20	2.30–2.72			
TNF-α (ng/mL)	Control	1.25	0.04	1.208–1.292	1.26	5.89	< 0.001^$^
	Disease	2.51	0.30	2.195–2.825			

Values are presented as mean ± standard deviation (SD). The symbol $ denotes statistically significant differences (*p* < 0.05), while # indicates non-significant differences (*p* ≥ 0.05).

*FBG* fasting blood glucose, *HOMA-IR* homeostatic model assessment of insulin resistance, *QUICKI* quantitative insulin sensitivity check index, *TC* total cholesterol, *TG* triglycerides, *VLDL-C* very low-density lipoprotein cholesterol, *LDL-C* low-density lipoprotein cholesterol, *HDL-C* high-density lipoprotein cholesterol, *ARI* atherogenic risk index, *CRI* coronary risk index, *CK-MB* creatine kinase-myocardial band, *IL-6* interleukin-6, *IL-1β* interleukin-1 beta, *TNF-α* tumor necrosis factor-alpha.

### Gross morphological examination of heart

**Fig. 6:**
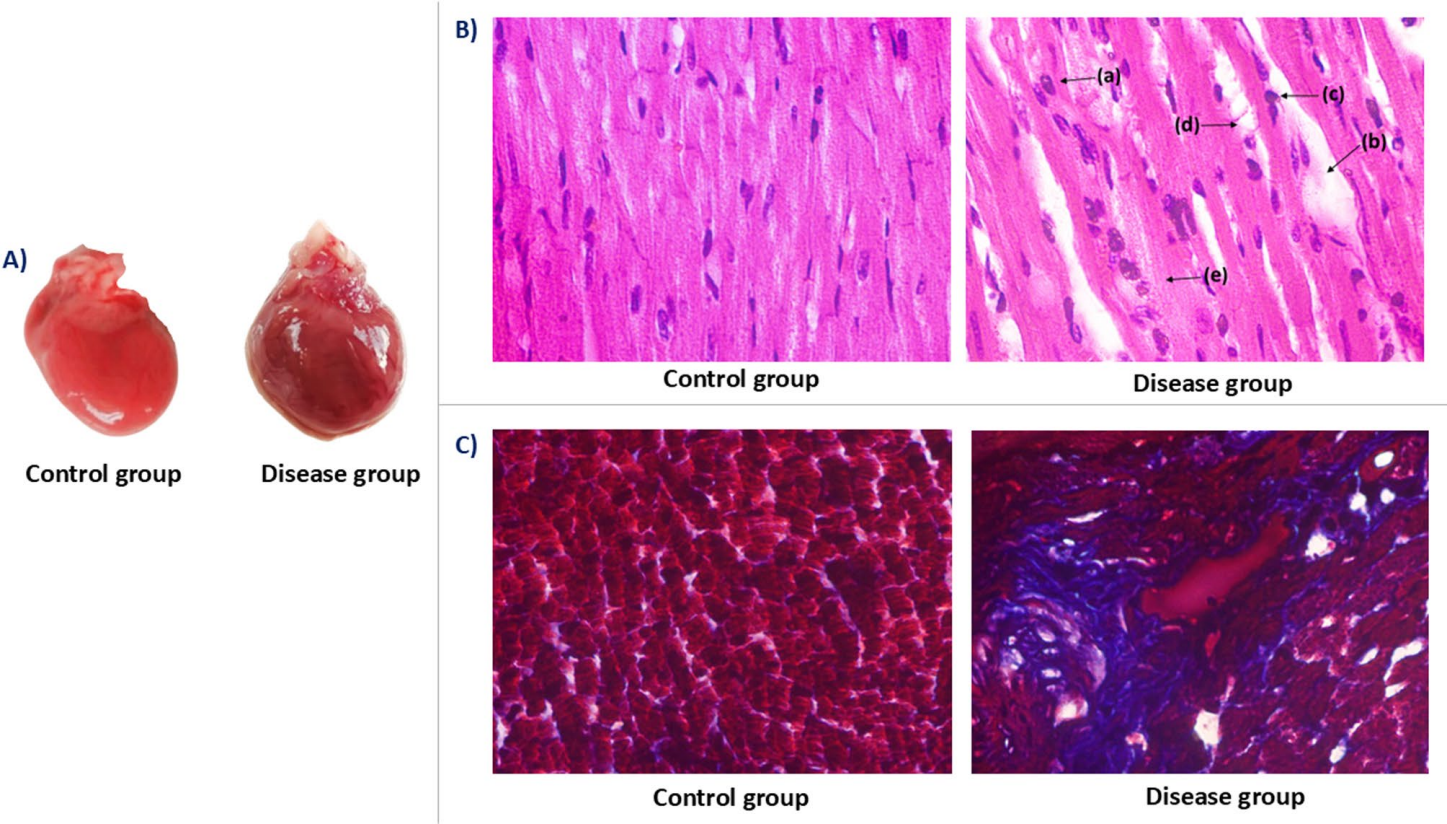
Cardiac gross and microscopic morphology. (**A**) Gross cardiac morphology. This panel displays representative gross images of isolated hearts. The left image shows a heart from the control group, characterized by a normal size, regular shape, smooth and glistening epicardial surface, and uniform reddish-pink coloration. The right image shows a heart from the disease group, which appears enlarged and more globular, with an irregular epicardial surface and patchy, dark red areas indicative of congestion. (**B**) Histopathological examination of cardiac tissues using hematoxylin and eosin stain (longitudinal section, 400X magnification). Representative photomicrographs illustrating the microscopic cellular architecture of cardiac tissue sections stained with Hematoxylin and Eosin (H&E). The left image depicts myocardial tissue from the control group, showing well-preserved architecture with cardiomyocytes arranged in parallel arrays, centrally located oval nuclei, intact myofibrillar striations, and unremarkable interstitial spaces. The right image, from the disease group, demonstrates marked structural alterations, including: (a) cardiomyocyte hypertrophy, (b) interstitial edema, (c) mononuclear inflammatory infiltrate, (d) cytoplasmic vacuolization, and (e) cardiomyocyte degeneration. (**C**) Histopathological examination of cardiac tissues using Masson’s Trichrome Stain (Transverse Section, 400X Magnification). Representative photomicrographs illustrating collagen deposition and fibrotic changes in the myocardium from control group and disease group rats, stained with Masson’s Trichrome (MT). The left image shows myocardial tissue from the control group, characterized by intact cardiomyocyte structure and minimal collagen deposition (red staining representing muscle, minimal blue representing collagen). The right image, from the disease group, highlights extensive interstitial and perivascular fibrosis, indicated by widespread blue staining of collagen fibers, demonstrating disrupted myocardial architecture and widened interstitial spaces.

Gross examination of heart from the control group revealed normal size and shape, characterized by a smooth, glistening epicardial surface and a uniform reddish-pink coloration. In contrast, heart from the disease group appeared enlarged and more globular relative to the control group. Additionally, the epicardial surface was irregular and exhibited patchy, dark red areas consistent with congestion (Fig. 6-A).

### Histopathological examination of cardiac tissues

Qualitative histological examination of cardiac tissues was performed on randomly selected animals from each group to assess the effects on cardiomyocyte architecture using Hematoxylin & Eosin (H&E) and Masson’s Trichrome (MT) staining under light microscopy at 400X magnification.

H&E-stained sections from the control group revealed well-preserved myocardial architecture. Cardiomyocytes were arranged in parallel arrays with centrally located, oval nuclei. Myofibrillar striations were intact, and the interstitial spaces were unremarkable, showing no evidence of edema or inflammatory infiltration.

In contrast, the disease group displayed marked structural alterations. The myocardial tissue showed myocyte hypertrophy, disorganization of cardiac fibers, and loss of parallel alignment. Several cardiomyocytes exhibited cytoplasmic vacuolization and nuclear enlargement. Additionally, focal interstitial edema and areas of mononuclear inflammatory cell infiltration were evident (Fig. 6B).

MT-stained sections further highlighted pathological changes in the disease group, including extensive interstitial and perivascular fibrosis. The normal architecture of the myocardium was disrupted, with widened interstitial spaces filled with collagen fibers. These fibrotic features were absent in the control group, which demonstrated intact cardiomyocyte structure and minimal collagen deposition (Fig. 6C).

## Discussion

In an effort to replicate the intricate interactions between cardiovascular risk factors seen in postmenopausal women, the current work successfully developed a multi-factorial cardiometabolic disease model in female Wistar rats by combining postmenopausal status, dietary obesity, and type 2 diabetic mellitus (T2DM). The metabolic and cardiovascular consequences linked to high-fat diet (HFD), streptozotocin (STZ)-nicotinamide-induced diabetes, and ovariectomy-induced estrogen depletion are accurately simulated by our model. This integrative approach is clinically relevant for studying disease mechanisms and therapeutic interventions in a high-risk population because it reflects the human clinical scenario, especially in postmenopausal women who frequently present with overlapping risk factors like obesity, insulin resistance, and hormone deficiency^57^.

One important biological change that has a big influence on metabolic and cardiovascular health is menopause. Weight gain, changed lipid profiles, and dysregulated energy metabolism are all consequences of estrogen deficiency, and they all raise the risk of metabolic diseases like obesity, insulin resistance, and cardiovascular disease. By regulating lipid metabolism, insulin sensitivity, and inflammatory responses, estrogen has cardioprotective effects and is essential for preserving metabolic balance^58^. Increased visceral adiposity, compromised glucose homeostasis, and elevated pro-inflammatory cytokine levels are all linked to loss of estrogen signaling, which puts postmenopausal women at risk for cardiometabolic syndrome. These effects have also been demonstrated in bilateral ovariectomy models, which show elevated levels of IL-6 and TNF-α, along with impaired myocardial function and increased cardiac fibrosis, mimicking the systemic inflammation seen in postmenopausal women with metabolic syndrome^59–61^.

Our study demonstrated a rapid and sustained increase in body weight in disease group animals following ovariectomy, with a significant increase observed by the fifth week (*p* = 0.005) and a highly significant increase (*p* < 0.001) in the subsequent weeks (Fig. 2). Yang et al.’s findings, which showed that ovarian hormone depletion causes early metabolic alterations, are consistent with this observation^62^. They found that rats that had bilateral ovariectomy gained more weight than rats that had sham surgery. It has been demonstrated that a lack of estrogen lowers energy expenditure, increases hunger through the dysregulation of hypothalamic neuropeptides, and encourages obesity by changing lipid metabolism to favor increased lipogenesis and decreased fatty acid oxidation^63^. Furthermore, insulin resistance and a pro-inflammatory state are encouraged by estrogen loss, which exacerbates metabolic dysfunction^64^. Similar findings have been reported in other ovariectomy-based models, where estrogen deficiency was associated with increased leptin, reduced adiponectin levels, and elevated markers of systemic inflammation, contributing to both metabolic and cardiovascular dysfunction^65,66^.

A known risk factor for cardiovascular disorders, obesity negatively impacts the anatomy and function of the heart. People who accumulate too much adipose tissue are more likely to develop heart failure and arrhythmias such as atrial fibrillation because of the increased myocardial mass, left ventricular hypertrophy, and altered cardiac electrophysiology that result from these conditions^67^. Moreover, dyslipidemia, which is marked by decreased levels of high-density lipoprotein cholesterol (HDL-C) and increased levels of triglycerides and low-density lipoprotein cholesterol (LDL-C), is closely linked to obesity and increases the risk of cardiovascular disease^68^. Dietary obesity was added as the second phase of our experimental model to investigate its synergistic impact on metabolic and cardiovascular parameters because of the substantial correlation between obesity and cardiovascular problems. Consistent with previous studies, our model showed dyslipidemia patterns reflective of the atherogenic lipid profile found in obese postmenopausal women, further validating its translational relevance (Fig. 3)^69,70^.

The absence of estrogen has been shown to potentiate the adverse effects of a high-fat diet, leading to more pronounced metabolic disturbances, including increased adiposity, impaired glucose metabolism, and heightened cardiovascular risk^71^. The stress on the cardiovascular system is further increased by the rising incidence of diabetes mellitus worldwide. Originally discovered as an antibiotic, STZ is frequently used to cause experimental diabetes because of its specific toxicity to beta cells in the pancreas. By entering beta cells through the GLUT2 transporter, STZ causes oxidative stress, mitochondrial malfunction, and DNA damage, all of which contribute to insulin insufficiency and hyperglycemia^72,73^. Nicotinamide is used in conjunction with STZ to mimic the pathophysiology of type 2 diabetes instead of type 1 diabetes and in part maintain beta-cell activity. Nicotinamide prevents excessive beta-cell death and permits residual insulin production via regulating poly(ADP-ribose) polymerase (PARP) activity^74^.

Due to beta-cell destruction, there is usually a brief spike in insulin release after STZ treatment, which causes hypoglycemia. Significant mortality may ensue from this early hypoglycemia if left untreated. To mitigate this risk, our study implemented a 5% glucose supplementation during the first 24 h post-injection, ensuring animal survival and metabolic stability^75^. Chronic hyperglycemia, insulin resistance, and dyslipidemia are the results of STZ-induced beta-cell failure, which mimics the metabolic abnormalities seen in human type 2 diabetes^76^.

The disease group in our study exhibited substantial metabolic and cardiovascular alterations compared to the control group. A significant increase in fasting blood glucose levels and body weight was observed, indicating the presence of metabolic dysfunction (Fig. 2). Electrophysiological disturbances observed in the disease group, when compared to the control group, were evident through significant alterations in electrocardiogram (ECG) parameters, including prolonged PR, QT, and corrected QT (QTc) intervals, along with a marked reduction in QRS amplitude (Fig. 4). The prolonged PR interval suggests delayed atrioventricular conduction, indicating possible first-degree heart block or impaired AV nodal function, which can compromise the coordination between atrial and ventricular contractions and predispose the heart to arrhythmias^77–79^. The prolongation of the QT and QTc intervals reflects delayed ventricular repolarization, increasing the risk for ventricular arrhythmias such as Torsades de Pointes and sudden cardiac death—making QTc prolongation a critical biomarker in preclinical screening of cardiotoxicity in drug development^80,81^. Additionally, the reduced QRS amplitude indicates impaired ventricular depolarization, potentially due to myocardial injury, edema, or fibrotic changes, which can compromise myocardial excitability and contractile function^82–84^. These findings are supported by other animal studies of bilateral ovariectomy models, which have demonstrated prolonged QTc intervals and reduced QRS amplitude as electrophysiological consequences of estrogen deficiency combined with metabolic dysregulation^70,85^.

Increased triglycerides, total cholesterol, and LDL-C levels, as well as a marked decrease in HDL-C levels, were indicative of dyslipidemia in the disease group. These alterations raise cardiovascular risk indices and aid in the development of atherosclerosis. Troponin T and creatine kinase-MB (CK-MB), two indicators of myocardial injury, were also markedly increased, suggesting myocardial damage and potential ischemic events (Fig. 5). Similar elevations in these cardiac biomarkers have been reported in other rodent models of ovariectomy and HFD-fed diabetic rats, confirming the predictive value of our model for detecting subclinical myocardial injury relevant to human cardiovascular disease^86,87^.

As evidenced by elevated insulin levels, HOMA-IR score and decreased QUICKI our model exhibited significant insulin resistance, a characteristic of type 2 diabetes mellitus (Fig. 2). Pro-inflammatory cytokines such as interleukin-6 (IL-6), interleukin-1β (IL-1β), and tumor necrosis factor-alpha (TNF-α) were significantly elevated in the disease group, indicating an inflammatory environment (Fig. 5). Chronic inflammation promotes endothelial dysfunction, oxidative stress, and atherosclerosis, serving as a major driver of metabolic syndrome and cardiovascular disease^88^. Together, our results highlight the harmful effects of dietary obesity, T2DM, and estrogen insufficiency on cardiovascular and metabolic health. These results are in line with prior studies, which have shown that disease group animals following ovariectomy and induction of diabetes mellitus, exhibit similar inflammatory cytokine profiles, correlating with myocardial inflammation and endothelial injury^60,89^.

Consistent with these findings, our gross and histopathological evaluations revealed significant cardiac structural damage in the disease group. Gross examination of hearts demonstrated enlargement and irregular epicardial surfaces with patchy congestion, reflecting possible myocardial stress and early pathological remodeling. Histological analysis corroborated these observations, showing cardiomyocyte hypertrophy, disorganization of myocardial fibers, cytoplasmic vacuolization, and nuclear enlargement. The presence of mononuclear inflammatory infiltration and interstitial edema further indicated active myocardial inflammation. Additionally, Masson’s Trichrome staining confirmed extensive interstitial and perivascular fibrosis in the disease group—features absent in control group—suggesting progressive cardiac remodeling and fibrotic deposition driven by chronic metabolic and hormonal insults. These structural and fibrotic alterations provide morphological evidence for the cardiac dysfunction observed electrophysiologically and biochemically, thereby validating the translational fidelity of our model (Fig. 6). Importantly, this model offers a valuable preclinical platform for evaluating cardioprotective and metabolic therapies specifically designed for postmenopausal women, who are often excluded from early-stage clinical trials. This model allows for the assessment of hormone replacement therapies (e.g., selective estrogen receptor modulators), lipid-lowering agents (e.g., statins, PCSK9 inhibitors), insulin sensitizers (e.g., metformin, thiazolidinediones), and anti-inflammatory drugs (e.g., IL-6 inhibitors), allowing researchers to explore drug efficacy in a physiologically relevant setting that mimics clinical complexity.

This study offers a significant advancement in translational cardiometabolic research by establishing a multifactorial preclinical model that closely mimics the complex interplay of risk factors observed in postmenopausal women. By integrating ovariectomy-induced estrogen deficiency, high-fat diet-induced obesity, and STZ-nicotinamide-induced type 2 diabetes, the model captures the hormonal, metabolic, and inflammatory perturbations that collectively drive cardiovascular dysfunction. The inclusion of diverse endpoints—ranging from metabolic parameters and pro-inflammatory cytokines to electrocardiographic alterations and cardiac biomarkers—enhances the model’s relevance and robustness. Importantly, this is one of the few studies to replicate overlapping clinical conditions within a single experimental framework, thereby providing a powerful and ethically optimized platform for investigating therapeutic strategies tailored to high-risk female populations often underrepresented in clinical trials.

This multi-factorial preclinical model provides a robust and translationally relevant platform for investigating the molecular and pathophysiological mechanisms underlying cardiometabolic diseases, particularly in postmenopausal women, a population often underrepresented in early-stage clinical trials. By recapitulating human-like disease progression, it is uniquely suited for testing novel therapeutic interventions targeting hormonal deficiencies, metabolic imbalances, and cardiovascular complications. The model also facilitates mechanistic studies of mitochondrial dysfunction, oxidative stress pathways, and endothelial biomarkers, and can be leveraged to evaluate pharmacological interventions, including hormone replacement therapies, metabolic modulators such as GLP-1 receptor agonists and SGLT2 inhibitors, anti-inflammatory agents, and cardioprotective strategies including PCSK9 inhibitors and statins. Overall, this model serves as a valuable bridge between preclinical and clinical research, supporting the development of personalized therapeutic strategies to improve cardiovascular outcomes in high-risk populations.

While this study offers a robust and translationally relevant model, certain carefully considered limitations were incorporated in alignment with ethical and logistical considerations. In compliance with the 3Rs (Replacement, Reduction, and Refinement) principle mandated by our Institutional Ethics Committee, we consciously limited the number of experimental animals to reduce unnecessary use, which precluded the inclusion of separate control groups for each intervention. This decision allowed us to simulate the cumulative impact of cardiometabolic risk factors while still maintaining scientific validity. However, we recognize the value of these controls and plan to incorporate them in future investigations to further strengthen the model and its translational relevance. Hormonal confirmation of estrogen depletion via serum estradiol levels was not performed—not as an oversight, but due to the high cost and limited availability of reliable estradiol assay kits at the time of study planning. Similarly, while blood pressure and vascular function are critical endpoints, the acquisition of non-invasive BP instrument was beyond the study’s allocated budget. Cardiac function assessments such as echocardiography, though valuable, were deferred to future studies where resources and infrastructure will allow more comprehensive physiological evaluations.

While the present study included sham-operated female rats on a standard diet as the sole control group, future investigations could incorporate additional experimental groups—such as ovariectomized-only rats on a standard diet, sham + high-fat diet, and ovariectomy + control diet—to better delineate the independent and combined effects of estrogen deficiency, dietary excess, and glycemic stress. Circulating estradiol levels and uterine weight, established biomarkers of estrogenic status, were not measured in this study, which limits direct confirmation of estrogen deprivation. Comprehensive functional cardiovascular assessments, including echocardiography and continuous blood pressure monitoring, were also not performed due to infrastructural constraints, and food intake was not systematically monitored, which reduces the ability to directly attribute body weight changes to consumption patterns. Although animal models inherently exhibit species-specific differences, the 25-week duration was strategically selected to capture long-term pathological changes while adhering to the ethical and practical constraints of preclinical research. Future extensions of this model may incorporate advanced mechanistic endpoints such as vascular reactivity, mitochondrial function, gene expression profiling, and echocardiographic parameters to further enhance its translational utility. Nevertheless, despite these considerations, this model provides a powerful and versatile platform for mechanistic investigations and therapeutic testing in postmenopausal cardiometabolic disease research.

Furthermore, our study establishes a robust experimental foundation for investigating the interconnected progression of obesity, diabetes, and estrogen deficiency in relation to cardiovascular disease. It provides new opportunities for exploring the pathophysiological underpinnings and testing novel interventions tailored to the unique cardiometabolic profile of postmenopausal women.

## Conclusion

An integrated preclinical model of cardiometabolic risk factors in female Wistar rats was developed effectively in this study, offering a translational platform for furthering cardiovascular research. This model successfully replicates the metabolic and cardiovascular dysfunctions frequently seen in postmenopausal women by including dietary obesity, streptozotocin-nicotinamide-induced type 2 diabetes mellitus (T2DM), and ovariectomy-induced estrogen depletion. Unlike traditional single-risk factor models, this multi-factorial approach better reflects the overlapping metabolic disturbances that contribute to cardiovascular disease progression in high-risk populations.

A high-fat diet exacerbated the progressive weight gain, metabolic dysregulation, and inflammatory alterations brought on by estrogen deprivation. Persistent hyperglycemia, insulin resistance, dyslipidemia, and electrophysiological abnormalities were the outcomes of inducing type 2 diabetes, which closely mirrored the pathology of ischemic heart disease. The substantial influence of these interrelated risk factors on cardiovascular health was demonstrated by the disease group’s notable changes in lipid profiles, pro-inflammatory cytokines, insulin resistance indicators, and ECG measurements. The significance of estrogen in maintaining metabolic balance and cardiac function was further supported by elevated Troponin T and CK-MB levels, which further suggested greater vulnerability to myocardial damage.

Ultimately, this study establishes a robust experimental foundation for evaluating the intricate interplay between diabetes, obesity, and estrogen deficiency in cardiovascular disease progression. Beyond deepening our understanding of disease mechanisms, this model sets the stage for targeted translational research efforts that cannot be adequately addressed by existing models, ultimately driving innovations in the prevention and treatment of cardiometabolic disorders.

## Data Availability

Data is provided within the manuscript.
